# A perspective on spaceflight associated neuro-ocular syndrome causation secondary to elevated venous sinus pressure

**DOI:** 10.1038/s41526-022-00188-6

**Published:** 2022-02-15

**Authors:** Grant Alexander Bateman, Alexander Robert Bateman

**Affiliations:** 1 Department of Medical Imaging, John Hunter Hospital, Newcastle, NSW Australia; 2 Newcastle University Faculty of Health, Callaghan Campus, Newcastle, NSW Australia; 3 School of Mechanical Engineering, University of New South Wales, Sydney, NSW Australia

**Keywords:** Refractive errors, Neuroscience

## Abstract

Spaceflight associated neuro-ocular syndrome (SANS) alters the vision of astronauts during long-duration spaceflights. There is controversy regarding SANS being similar to patients with idiopathic intracranial hypertension (IIH). IIH has been shown to be due to an elevation in venous sinus pressure. The literature suggests an increase in jugular vein pressure secondary to a headward shift of fluid occurs in SANS but this may not be enough to significantly alter the intracranial pressure (ICP). The literature regarding cardiac output and cerebral blood flow (CBF) in long-duration spaceflight is contradictory, however, more recent data suggests increased flow. Recent modelling has shown that an increase in CBF can significantly increase sinus pressure. The purpose of the present paper is to review the SANS vascular dynamics literature and through mathematical modelling suggest the possible underlying cause of SANS as an elevation in venous sinus pressure, secondary to the redistribution of fluids towards the head, together with a significant increase in pressure drop across the venous system related to the CBF.

## Introduction

Spaceflight associated neuro-ocular syndrome (SANS) refers to the pathological effects of long-term microgravity on the eyes and orbital physiology of astronauts. The clinical manifestations of SANS include unilateral and bilateral optic disc oedema, globe flattening, choroidal and retinal folds, hyperopic refractive error shifts, and focal areas of ischaemic retina^1^. Although 15% of astronauts have clinically significant optic disc oedema, subclinical oedema has been detected in many astronauts returning from long-duration flights^1^.

It has been suggested the findings in SANS may be similar to terrestrial idiopathic intracranial hypertension (IIH)^1^, however, others disagree^2^. IIH is characterised by an increased intracranial pressure (ICP) in the absence of parenchymal brain lesions, vascular malformations, hydrocephalus or central nervous system infection^3^. To diagnose IIH, a cerebrospinal fluid (CSF) pressure above 25 cm H_2_O (18.4 mmHg) is required according to the published guidelines^4^. A patient is suggested of having IIH if they have 3 out of 4 additional imaging criteria i.e. compression of the pituitary, flattening of the posterior globe, distention of the optic nerve sheath diameter (ONSD) or transverse venous sinus stenosis^4^. In long flight astronauts, globe flattening was seen in 26%, optic nerve protrusion (optic disc oedema) in 15% and pituitary flattening in 11%^5^. Seven astronauts with globe flattening had a mean ONSD of 7.2 mm compared to 20 without globe flattening who had a mean ONSD of 5.8 mm (*p* = 0.01)^5^. Although it should be acknowledged there was no pre-flight control data, the ONSD findings in the seven with orbital flattening are above the level associated with IIH^5^. Thus, three of the imaging criteria for IIH are found in long flight veterans. Three of the astronauts underwent post-flight lumbar puncture showing pressures of 23, 28 and 29 cm H_2_O^5^ (inflight data is not available), with 2 of these pressures being in the diagnostic range for IIH^4^. A counterpoint paper suggests there is no evidence of ONSD enlargement secondary to spaceflight^6^ but the study was underpowered and had only one individual who developed SANS. This individual had an increase in ONSD in the range suggestive of IIH.

It can be seen that by utilising the revised IIH criteria, there are some astronauts who would fulfil the criteria for IIH. The purpose of this perspective is to review the literature in SANS with respect to the blood and CSF dynamics. Following this, computational fluid dynamics modelling will be utilised to suggest the possible causes of SANS in astronauts undergoing long-duration space flight and to suggest a vascular aetiology for SANS which is similar to IIH.

## Discussion

There are similarities between SANS and IIH, however, there are also differences. Terrestrial symptoms of IIH include headache and pulsatile tinnitus, but astronauts with SANS do not complain of these symptoms^7^. IIH presents more often as bilateral eye changes, predominately in women, but SANS has a higher asymmetric or unilateral presentation and is more common in men^1^. Transient visual obscurations or diplopia secondary to a non-localising sixth nerve palsy have never been reported in astronauts with SANS, unlike IIH^1^. Despite these differences, there remain enough similarities to suggest some commonality may exist in the pathophysiology of both.

The main criteria for IIH is an elevation in the ICP which is modelled using Davson’s equation

1 $${{{\mathrm{ICP}}}} = {{{\mathrm{FR}}}}_{{{{\mathrm{CSF}}}}}\;{{{\mathrm{x}}}}\;{{{R}}}_{{{{\mathrm{out}}}}} + {{{\mathrm{SSS}}}}_{{{\mathrm{p}}}}$$

where FR_CSF_ is the CSF formation rate, *R*_out_ is the CSF outflow resistance and SSS_p_ is the superior sagittal sinus pressure. The sagittal sinus pressure is analogous to Ohm’s law, i.e. pressure is the product of the outflow resistance and blood flow through the outflow plus the jugular bulb pressure^8^. So Eq. (1) can be expanded to

2 $${{{\mathrm{ICP}}}} = {{{\mathrm{FR}}}}_{{{{\mathrm{CSF}}}}}\;{{{\mathrm{x}}}}\;{{{R}}}_{{{{\mathrm{out}}}}} + {{{\mathrm{TCBF}}}}\;{{{\mathrm{x}}}}\;{{{R}}}_{{{{\mathrm{ven}}}}} + {{{\mathrm{JBP}}}}$$

where TCBF is the total blood flow leaving the capillaries to enter the venous system, *R*_ven_ is the venous outflow resistance from the sagittal sinus to the jugular bulbs and JBP is the jugular bulb pressure. The CSF formation rate is normal in IIH and decreases as the ICP increases^9^. The pressure gradient between the CSF and sagittal sinus was found to low-normal at 2.34 mmHg in one paper in IIH^10^ and 2.7 mmHg in another^11^. Rearranging Eq. (1) by subtraction, the ICP to sagittal sinus pressure gradient is equal to the FR_csf_ x R_out_, and if this term is normal, then the elevation in ICP in IIH can only be due to elevated venous pressure. This discussion tends to validate the original assertion made by Karahalios et al. i.e. “an elevated venous pressure is the universal mechanism underlying IIH”^12^. Is a venous pressure elevation also the universal cause of SANS?

The normal ICP is 15.6 cm H_2_O or 11.5 mmHg in the lateral decubitus position in adults between 20 and 49 years^13^. The criteria for IIH is an ICP of 18.4 mmHg (or 25 cm H_2_O)^4^. Thus a prolonged increase in venous sinus pressure of approximately 7 mmHg would be required if SANS were analogous to IIH. As previously described, the venous sinus pressure depends on the combination of the jugular bulb pressure, venous outflow resistance and the total blood flow passing through the venous system. In adults with IIH, 71% are obese^14^. Obesity raises the jugular bulb pressure by up to 20 mmHg^15^. Up to 90% of adults with IIH have been found to have outflow stenosis mostly sited at the middle of the transverse sinus, raising the venous resistance and therefore the venous pressure^16^. Sixty-six per cent of the increase in ICP in IIH is due to venous stenosis and 34% is due to obesity^17^. Astronauts are not obese^1^. A recent paper comparing the venous sinus volumes, both before and after spaceflight, showed a significant increase in the size of the superior sagittal, transverse and sigmoid sinuses (not stenosis) in those who developed SANS, compared to those who did not^18^. In another paper, in three long-duration astronauts with borderline or elevated CSF opening pressures, the MRV images were reported as normal with no evidence of stenosis or thrombosis^19^.

Therefore, if the physiology of SANS is similar to IIH, there must be other factors elevating the venous sinus pressure.

### Current hypotheses regarding SANS aetiology

The main hypothesis regarding the cause of SANS centres on a rise in ICP due to the cephalad fluid shifts occurring during long-duration spaceflight^1^. Similar to obesity, this would have the effect of directly raising the venous sinus pressure. A second hypothesis suggests that CSF accumulates in the optic nerve sheath due to a one-way valve mechanism^20^. This hypothesis would explain the eye findings but not the elevated ICP or pituitary compression found in some astronauts. A third hypothesis suggests that SANS is due to upward shift of the brain putting tension on the optic nerve sheath^21^. This hypothesis is not necessarily mutually exclusive of hypothesis 1 but of itself does not explain the elevation in ICP.

### Cephalad fluid shift hypothesis

Ideally, all hypotheses would be tested on astronauts whilst in space, during a long-duration flight. However, the numbers of subjects available are limited, an astronaut’s time in space is precious and the technique used must be achievable in space and must match the expertise of the subjects. For example, performing magnetic resonance imaging (MRI) or lumbar punctures on board the International Space Station is not feasible. Thus, ground-based space analogues are utilised. Analogues such as supine bed rest, head-down tilt bed rest (HDTBR), wet and dry immersion, lower-extremity limb suspension and parabolic flight have been tried^22^.

One space-based study used ultrasound-guided jugular vein compression. The terrestrial jugular venous pressure varies between the two-thirds of the day one spends upright and the one-third supine. If one weighted the pressures found (66% of the upright figure plus 33% of the supine) then the 24-h weighted average jugular venous pressure was found to be 9.2 mmHg when on the ground and increased by 6.6 mmHg at day 150 during long term weightlessness^23^. Note that this is very close to the 7 mmHg increase in pressure required to trigger terrestrial IIH. Head-down tilt experiments, similar to the jugular vein compression study, indicate that a headward redistribution of fluid brings about a dilatation of the jugular veins with some evidence of increased ICP^24^. Strict head-down tilt experiments can cause retinal thickening and chorioretinal folds but no changes in refractive error or perimetry developed^25^. This suggests the headward shift is able to produce a syndrome similar to mild SANS.

### Cardiac output during long-term spaceflight and HDTBR

During spaceflight, the cardiac output (CO) appears to depend on which technique is used. Two studies using ultrasound echocardiography observed a decrease in stroke volume and CO during flight^26,27^. Hughson et al. used the continuous finger blood pressure contour technique and found no changes in stroke volume^28^. However, Norsk et al. found significant increases in stroke volume and CO of as much as 35 and 41%, respectively, between 3 and 6 months of flight on the ISS^29^. A foreign gas rebreathing technique was used^29^. Hughson et al. later measured CO and stroke volume by the same method as Norsk et al. and found similar increases^30^. They concluded that their previously unchanged stroke volume and CO estimations had been incorrect^30^. The increase in CO of 41–56% after months^29,30^ is higher than the increase measured by the same technique i.e.18–26% reported from shorter week long-Space Shuttle missions^31–33^. Thus, long durations in space produce higher stroke volumes and COs than a short duration. Norsk comments, both foreign gas rebreathing and ultrasound techniques have been validated thoroughly and multiple times against invasive more direct, gold standard techniques. The foreign gas rebreathing method is observer-independent and obtains data over a longer time period (20 s) than the ultrasound Doppler (three cardiac cycles) and that the breathing frequency and depth are controlled and kept constant during the measurements. Thus, he suggests the gas technique is probably correct^34^. The cerebral blood flow (CBF) at rest is 15–20% of the total CO^35^ and the renal blood flow is 20% of CO^36^, In a low powered study of only 5 individuals, the renal plasma flows increased by 17% at 1 week in orbit but this was not significantly different to preflight values^37^. If a larger cohort had found this difference to be significant then this would be similar to the 18–26% increase in CO found by Norsk^31–33^. The CO must all pass through a capillary bed to return to the heart. If the CO is increased by 41% then the renal and CBFs cannot be normal.

### CBF in space flight and HDTBR

Similar to the findings in CO, the CBF findings seem to depend on the technique being used. A 5-day flight of a Rhesus monkey on the Cosmos 1514 biosatellite fitted with a carotid flow sensor showed a sustained 8 cm/s increase in blood velocity^38^. Impedance rheoencephalography has shown increased CBF both during and immediately after flight^39–43^. Transcranial Doppler (TCD) showed an increase in straight sinus velocity in 9/13 astronauts, with flow velocities ranging from 30–47 cm/sec and the normal range being 14–28 cm/sec^44^.

The transcranial Doppler of the middle cerebral arterial literature in spaceflight is contradictory. Some studies suggest an increase in cerebral blood velocity^45–48^, some unchanged^49,50^ and some decreased flow^51,52^. The divergence between the previously described measures of CBF and the transcranial Doppler velocity is striking. The original validation studies for TCD showed the correlation between the arterial velocity and the gold standard for CBF (xenon 133 CT) was weak at *r* = 0.42^53^. This is because the CBF depends on both the velocity of the blood and the cross-sectional area of the vessel. If the middle cerebral artery is constricted, the CBF will fall but the velocity will increase and in reverse, the velocity will drop but the flow increases when the diameter increases^54^. The middle cerebral artery lumen can increase in size by 45% by altering carbon dioxide (CO_2)_ levels from low-normal to high-normal^55^. Theoretically, if the CBF increased purely due to an increase in diameter, then the TCD could underestimate the effect by 45%. Indeed, Coverdale et al. found a change in middle cerebral artery cross-sectional area during acute hypercapnia resulted in a CBF underestimation by TCD of 18%^56^.

A recent study has shown a significant increase in the middle cerebral vein velocity on day 150 of spaceflight and ascribed this to a reduction in the vein diameter with a resulting normal venous flow volume^57^. However, the 84% increase in velocity found would require a 46% reduction in vein diameter to give a normal blood flow volume^57^. This would require the ICP to approach cerebral perfusion pressure^58^. This is because as the ICP increases the collapse of veins begins close to their outflow with the more proximal sections (where TCD measures velocity) being increased in size^59^. Only once the pressure reaches perfusion pressure will the whole vein collapse. No one suggests ICP approaches perfusion pressure in space as this would be lethal. Roberts et al. found some elevation of the brain and crowding of the sulci over the vertex and suggested this could lead to venous narrowing or arachnoid granulation obstruction. However, static pressures are equally distributed throughout a water bath and the reduction in the sulci was mild and unlikely to be water tight^58^. If the TCD vein diameter is normal then an increase in velocity must indicate an increase in blood flow volume.

Similar to the TCD findings, HDTBR studies tend to show a reduced CBF. This is perhaps not surprising as HDTBR also produces a reduction in cardiac stroke volume and CO^60^. There appears to be a distinct physiological difference between spaceflight and HDTBR. Thirteen days of space flight in a mouse model led to both a decrease in myogenic vasoconstrictor tone and an increase in arterial distention, leading to a 20% increase in resting cross-sectional area^61^. This would be consistent with lower vascular resistance and higher CBF during space flight^61^. This compared to control head-down tilt mice where there was no effect on vasoconstriction, passive pressure-diameter response or maximal diameter^61^. Using an MRI arterial spin labelling (ASL) technique, 30 days of head-down tilt plus increased CO_2_, demonstrated the expected decrease in CBF^62^. However, the five participants who developed optic disc oedema had higher CBF (approaching the baseline levels) compared with the non-oedema group^62^. This suggests that variations in vasoreactivity and autoregulatory responses to CO_2_ may play a role in SANS development^62^. Acute hypercapnia increases the CBF but the effect is short-lived being corrected within a few hours^63^. However, there is a delayed, more long-lasting vasodilatation occurring with chronic CO_2_ elevation which appears to be genetically mediated^63^. In chronic obstructive pulmonary disease, patients retain CO_2_. A reduction in the CO_2_ by non-invasive positive pressure ventilation reduced the middle cerebral artery TCD velocity by 23% without affecting the oxygen levels^64^. It should be noted TCD may have underestimated this effect. In a series of patients undergoing ASL MRI, those found to have an elevated CO_2_ had a grey matter CBF double the patients with CO_2_ in the normal range^65^. The effect was not statistically different between acute and chronic hypercapnia^65^. Overall, there was a 6.7% increase in CBF per 1-mmHg rise in partial pressure of CO_2_^65^. Interestingly, women tended to be more resistant to the effect of CO_2_ than men^65^ which may point to a reason why men are more prone to develop SANS than women.

**Fig. 1:**
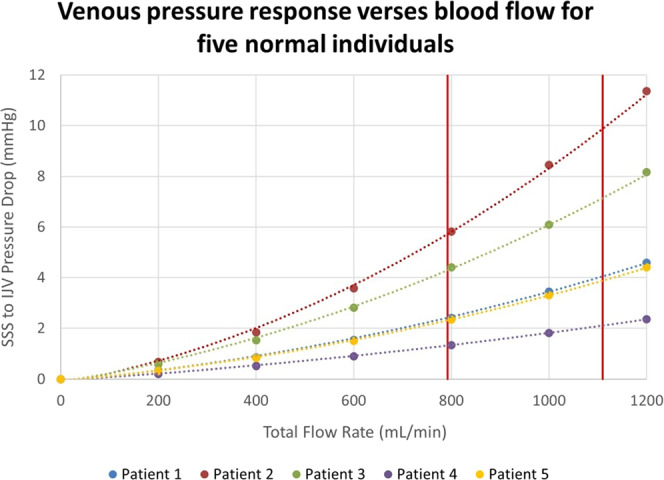
Venous pressure vs. blood flow. A graph of the venous pressure response vs. blood flow for the five patients modelled in reference^69^. The two vertical lines represent the normal flow rate at middle age on the left (792 mls/min) and a 40% increase in flow on the right (1110 mls/min). Note the pressure drop across the venous system is close to the normal figure of 2.5 mmHg at a normal flow rate for 3 of the 5 patients but elevated in 2. At a flow rate of 1110 mls/min there is a wide variation in response meaning there is a variable risk for the development of SANS if this condition is analogous to IIH. IVJ internal jugular vein, SSS superior sagittal sinus.

Thus, an increase in CO and CBF in long-term space flight may be related to CO_2_ in long-duration spaceflight. Technical constraints maintain carbon dioxide levels at nearly 10 times earth normal levels^1^. Air convection is also significantly reduced in microgravity and pockets of CO_2_ may develop around the nose and mouth of astronauts. At an ambient partial pressure of CO_2_ of 3.8 mmHg during flight, the expired CO_2_ of a cohort of astronauts was 6.1 mmHg higher than pre-flight levels^66^. There is a 6–6.7% increase in CBF for each 1-mmHg rise in partial pressure of CO_2_^65,67^. This indicates the CBF could be increased by the 41% increase in CO noted by Norsk et al.^29^. Forty-five per cent of children with IIH have cerebral hyperaemia^17^. A study in patients with IIH showed a 56% increase in CO_2_ led to a 166% increase in superior sagittal sinus pressure^68^ indicating the effect of CO_2_ on sinus pressure is non-linear. In a modelling study, the increase in blood returning through the venous system significantly increased the pressure drop from the sagittal sinus to the jugular bulbs because the pressure response to CBF of the venous system was quadratic in nature^69^. The cause of the greater than the linear response of the venous system to blood flow is due to the development of rotational or vortex flow induced by the unusual geometry. Could an increased blood flow in long-term space flight increase the venous pressure is SANS? The normal arterial inflow for a group of average age 43 years is 792 mls/min^70^. A 40% increase in arterial inflow secondary to the elevated CO_2_ would lead to a mean arterial inflow of 1110 mls/min. If we were to recruit the 5 normal individuals who had their normal venous outflow modelled with computational fluid dynamics (CFD) in our original paper^69^, and place them in orbit for 6 months, then we can see from Fig. 1 that there would be a variable increase in the venous pressure. The normal pressure drop across the cerebral venous system is 2.5 mmHg in middle age^17^. IIH needs an increase in venous pressure of 7 mmHg to develop. We can see in Fig. 1 that under hyperaemic conditions patient 4 would have no significant increase in pressure drop across the venous system compared to normal and presumably not be at high risk for IIH. Patients 5 and 1 would have pressure drops of 1.7 and 1.8 mmHg, respectively, above the normal figure, giving an increase in sinus pressure overall (once the 6.6 mmHg increase in jugular vein pressure from the headward fluid shift is added) of 8.3 and 8.4 mmHg and be at moderate risk of IIH and perhaps SANS. Patients 3 and 2 would have sinus pressure drops of 4.5 and 7.3 mmHg above normal, giving total sinus pressure increases of 11.1 and 13.9 mmHg and presumably be at a high risk of developing IIH and possibly SANS. Fifteen per cent of long-duration space flight astronauts have SANS and 20% have choroidal folds^1^, perhaps correlating with the 20% of our venous blood flow models which have suggested a large increase in pressure with increased CBF. The fivefold variation in the pressure response to the same increase in blood flow noted suggests that there is a wide variation in response to cerebral hyperaemia in normal individuals. Therefore, there may be some utility in performing CFD modelling studies on the venous outflow of prospective long-duration astronauts to try and stratify the risk of developing SANS.

## Methods

Initially, a literature search was undertaken in PUBMED looking for all research papers written in the English language with the search criteria “spaceflight associated neuro-ocular syndrome” or “vision impairment intracranial pressure”. Ninety-eight responses were obtained. Papers were chosen for review on the basis of discussing the CSF or blood flow dynamics. After the review, mathematical estimation of venous pressure in long term space flight was undertaken to utilise the computational fluid dynamic modelling undertaken by one of the authors (A.B.). The modelling data consists of 5 computational fluid dynamic models of the cerebral venous outflow from the sagittal sinus to the jugular veins obtained from the CT venogram images of patients who did not have evidence of cerebral disease, please see the original modelling study for further details^69^. Some of this data has been previously published^69^. Informed consent was obtained from all five patients who had their CT venogram data modelled to provide Fig. 1. The study original modelling study was approved by the Hunter New England Area Health Ethics Committee, therefore, the original study has been performed in accordance with the ethical standards laid down in the 1964 Declaration of Helsinki. The authorisation number HREG/10/NHE/132 was issued.

### Reporting summary

Further information on research design is available in the Nature Research Reporting Summary linked to this article.

## Supplementary information

Reporting Summary

## Data Availability

The data that support the findings of this study are available from the corresponding author upon reasonable request.
